# Upregulation of the hypothalamo-neurohypophysial system and activation of vasopressin neurones attenuates hyperalgesia in a neuropathic pain model rat

**DOI:** 10.1038/s41598-022-17477-5

**Published:** 2022-07-29

**Authors:** Kazuhiko Baba, Makoto Kawasaki, Haruki Nishimura, Hitoshi Suzuki, Takanori Matsuura, Naofumi Ikeda, Teruaki Fujitani, Yoshiaki Yamanaka, Manabu Tsukamoto, Hideo Ohnishi, Mitsuhiro Yoshimura, Takashi Maruyama, Kenya Sanada, Satomi Sonoda, Kazuaki Nishimura, Kentaro Tanaka, Tatsushi Onaka, Yoichi Ueta, Akinori Sakai

**Affiliations:** 1 Department of Orthopaedic Surgery, School of Medicine, University of Occupational and Environmental Health, 1-1 Iseigaoka, Yahatanishi-ku, Kitakyushu, 807-8555 Japan; 2 Department of Physiology, School of Medicine, University of Occupational and Environmental Health, Kitakyushu, 807-8555 Japan; 3 Division of Brain and Neurophysiology, Department of Physiology, Jichi Medical University, Shimotsuke, 329-0498 Japan

**Keywords:** Neuroscience, Physiology

## Abstract

Arginine vasopressin (AVP) is a hypothalamic neurosecretory hormone well known as an antidiuretic, and recently reported to be involved in pain modulation. The expression kinetics of AVP and its potential involvement in the descending pain modulation system (DPMS) in neuropathic pain (NP) remains unclear. We investigated AVP expression and its effects on mechanical and thermal nociceptive thresholds using a unilateral spinal nerve ligation (SNL) model. All rats with SNL developed NP. Intensities of enhanced green fluorescent protein (eGFP) in the supraoptic and paraventricular nuclei, median eminence, and posterior pituitary were significantly increased at 7 and 14 days post-SNL in AVP-eGFP rats. In situ hybridisation histochemistry revealed significantly increased *AVP* mRNA expression at 14 days post-SNL compared with the sham control group. The chemogenetic activation of AVP neurones significantly attenuated mechanical and thermal hyperalgesia with elevated plasma AVP concentration. These analgesic effects were suppressed by pre-administration with V1a receptor antagonist. AVP neurones increased the neuronal activity of serotonergic dorsal raphe, noradrenergic locus coeruleus, and inhibitory interneurones in the spinal dorsal horn. These results suggest that the hypothalamo-neurohypophysial system of AVP is upregulated in NP and activated endogenous AVP exerts analgesic effects via the V1a receptors. AVP neurones may activate the DPMS.

## Introduction

Arginine vasopressin (AVP), a hypothalamic neurosecretory hormone, is synthesised in the supraoptic nucleus (SON) and paraventricular nucleus (PVN) of the hypothalamus and secreted from the posterior pituitary (PP) to the systemic circulation^1^. AVP plays important roles in body fluid balance, blood pressure regulation, sodium homeostasis, and kidney function^2^. AVP also exhibits analgesic effects with its systemic, intracerebroventricular (i.c.v.), intrathecal (i.t.), and subcutaneous (s.c.) administration in rodents^3–7^. Moreover, intranasal AVP administration effectively treats headaches and pain after orthopaedic surgery in humans^8,9^.

The International Association for the Study of Pain defines neuropathic pain (NP) as pain caused by a lesion or disease of the somatosensory nervous system, NP is commonly classified as a form of chronic pain that has a major impact on quality of life^10^. Approximately 15–50% of patients with NP suffer from allodynia, defined as a pain due to a stimulus that does not typically cause pain, and hyperalgesia, described as an increased sensitivity to feeling pain^11^. However, NP is intractable to pharmacological treatment; thus, it is necessary to elucidate its complex pathology^12^. The dysfunction of the descending pain modulation system (DPMS) is one of the targets of chronic pain^13^. The serotonergic and noradrenergic pathways of the DPMS modulate pain in the spinal dorsal horn^14^. The serotonergic pathway includes the periaqueductal gray (PAG), dorsal raphe nucleus (DR), rostral ventromedial medulla (RVM), and nucleus raphe magnus (NRM)^13,15,16^. The noradrenergic pathway consists of the locus coeruleus (LC) and dorsolateral pontine tegmentum (DLPT)^13,17^. DPMS is implicated in the development and maintenance of NP^13,18^. To our knowledge, whether AVP neurones activate the DPMS has not been investigated.

The expression kinetics of the AVP system in NP have not been previously studied. We have previously reported increased endogenous AVP expression in formalin-induced inflammation, adjuvant-induced arthritis (AA), acute monoarthritis, and knee osteoarthritis (OA) in AVP-enhanced green fluorescent protein (eGFP) transgenic (Tg) rats^19–22^. In AVP-eGFP Tg rats, the activity of AVP neurones can be visualised in vivo and ex vivo without the use of immunohistochemistry by AVP and Fos^23^. Specifically, the expression level of AVP can be evaluated by measuring the fluorescence intensity of eGFP and counting the number of eGFP-positive neurones. However, it remains unclear whether increased endogenous AVP has an analgesic effect in various nociceptive models. Therefore, we established a Tg rat line that expresses human muscarinic acetylcholine receptors (hM3Dq) and mCherry fluorescence specifically in AVP neurones^24^. Using this Tg rat model, we reported a significant increase in plasma AVP and a significant decrease in food and water intake and urine volume after intraperitoneal (i.p.) administration of clozapine-N-oxide (CNO), an agonist of hM3Dq^24^. This AVP-hM3Dq-mCherry Tg rat model enables us to investigate the specific effects of endogenous AVP activated by CNO, which cannot be mimicked by administering exogenous AVP. In the present study, we evaluated the expression of endogenous AVP in a unilateral spinal nerve ligation (SNL) model and investigated whether enhanced endogenous AVP has an analgesic effect and activates the DPMS.

## Results

### SNL induces mechanical and thermal hyperalgesia and increases the number of Iba-1 and GFAP-positive cells in the L5 ipsilateral dorsal horn

**Figure 1:**
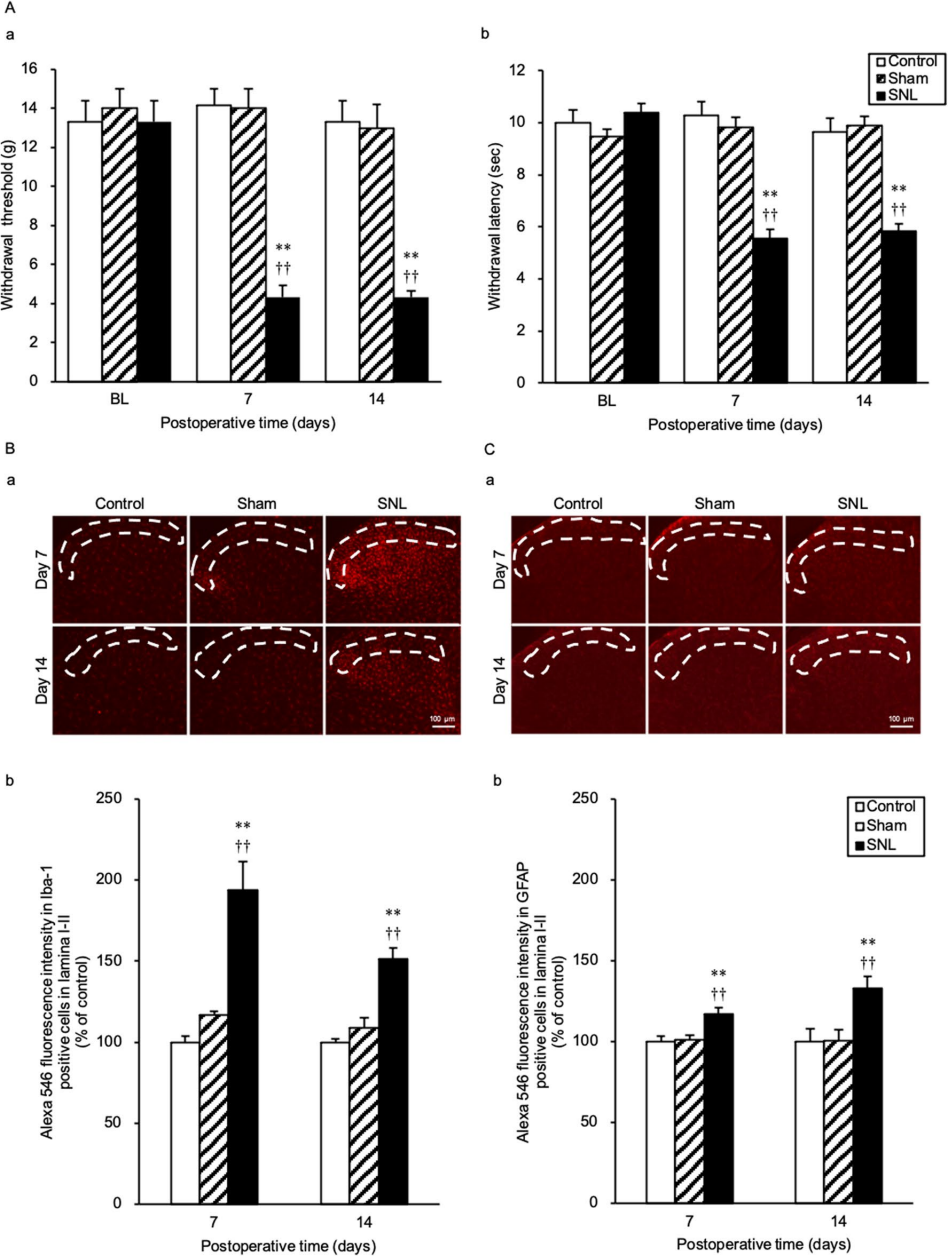
SNL induces mechanical and thermal hyperalgesia and increases the number of Iba-1 and GFAP-positive cells in the L5 ipsilateral dorsal horn at day 7 and 14 after SNL in AVP-eGFP Tg rats. (**A**) The change of mechanical and thermal nociceptive threshold. (**a**) Mean values of withdrawal thresholds to von Frey hair stimulation of the ipsilateral hind paw at the baseline (BL) on days 7 and 14 after SNL operation in Control, Sham surgery, and SNL rats. (**b**) Mean value of withdrawal latency to thermal stimulation of the ipsilateral hind paw at the BL and days 7 and 14 after SNL operation. (**B**) Change in ionised calcium-binding adapter molecule 1(Iba-1)-positive cells in the lumbar segment 5 (L5) ipsilateral dorsal horn. (**a**,**b**) Digital image of Iba-1-positive cells in the L5 ipsilateral dorsal horn and the average Alexa 546 fluorescence intensity in Iba-1 positive cells in laminae I–II. (**C**) Change in glial fibrillary acidic protein (GFAP)-positive cells. (**a**,**b**) Digital image of GFAP-positive cells in the L5 ipsilateral dorsal horn and the average Alexa 546 fluorescence intensity in GFAP-positive cells in laminae I–II. Sections were obtained from Control, Sham, and SNL groups on days 7 and 14 after SNL surgery. The area surrounded by a white dotted line indicates the layer of laminae I–II. Data are presented as the mean ± SEM (one-way ANOVA). *n* = 5–6 per group. **p < 0.01 compared with each Control experiment. ^††^*p* < 0.01 compared to Sham group.

Hyperalgesia occurred in all SNL-treated AVP-eGFP Tg rats. The withdrawal threshold against mechanical stimuli was significantly decreased in the SNL group compared with the Control and Sham groups at day 7 [F(2,14) = 49.61; SNL vs Control, *p* < 0.001; SNL vs Sham, *p* < 0.001] and at day 14 [F(2,14) = 31.95; SNL vs Control, *p* < 0.001; SNL vs Sham, *p* < 0.001] after the SNL (Fig. 1Aa). Withdrawal latency against thermal stimuli was significantly shortened in the SNL group compared with the Control and Sham groups at day 7 [F(2,14) = 38.09; SNL vs Control, *p* < 0.001; SNL vs Sham, *p* < 0.001] and day 14 [F(2,14) = 30.85; SNL vs Control, *p* < 0.001; SNL vs Sham, *p* < 0.001] after the SNL (Fig. 1Ab).

The fluorescence intensity of microglial Iba-1 was significantly higher in the SNL group than that in the Control and Sham groups at day 7 [F(2,14) = 20.46; SNL vs Control, *p* < 0.001; SNL vs Sham, *p* = 0.001] and at day 14 [F(2,14) = 26.16; SNL vs Control, *p* < 0.001; SNL vs Sham, *p* < 0.001] after the SNL (Fig. 1Ba,b). The fluorescence intensity of astrocytic GFAP was significantly higher in the SNL group than that in the Control and Sham groups at day 7 [F(2,14) = 7.46; SNL vs Control, *p* = 0.011; SNL vs Sham, *p* = 0.021] and day 14 [F(2,14) = 6.81; SNL vs Control, *p* = 0.017; SNL vs Sham, *p* = 0.026] after the SNL (Fig. 1Ca,b).

### SNL increases intensities of AVP-eGFP fluorescence in SON, PVN, median eminence, and PP

**Figure 2:**
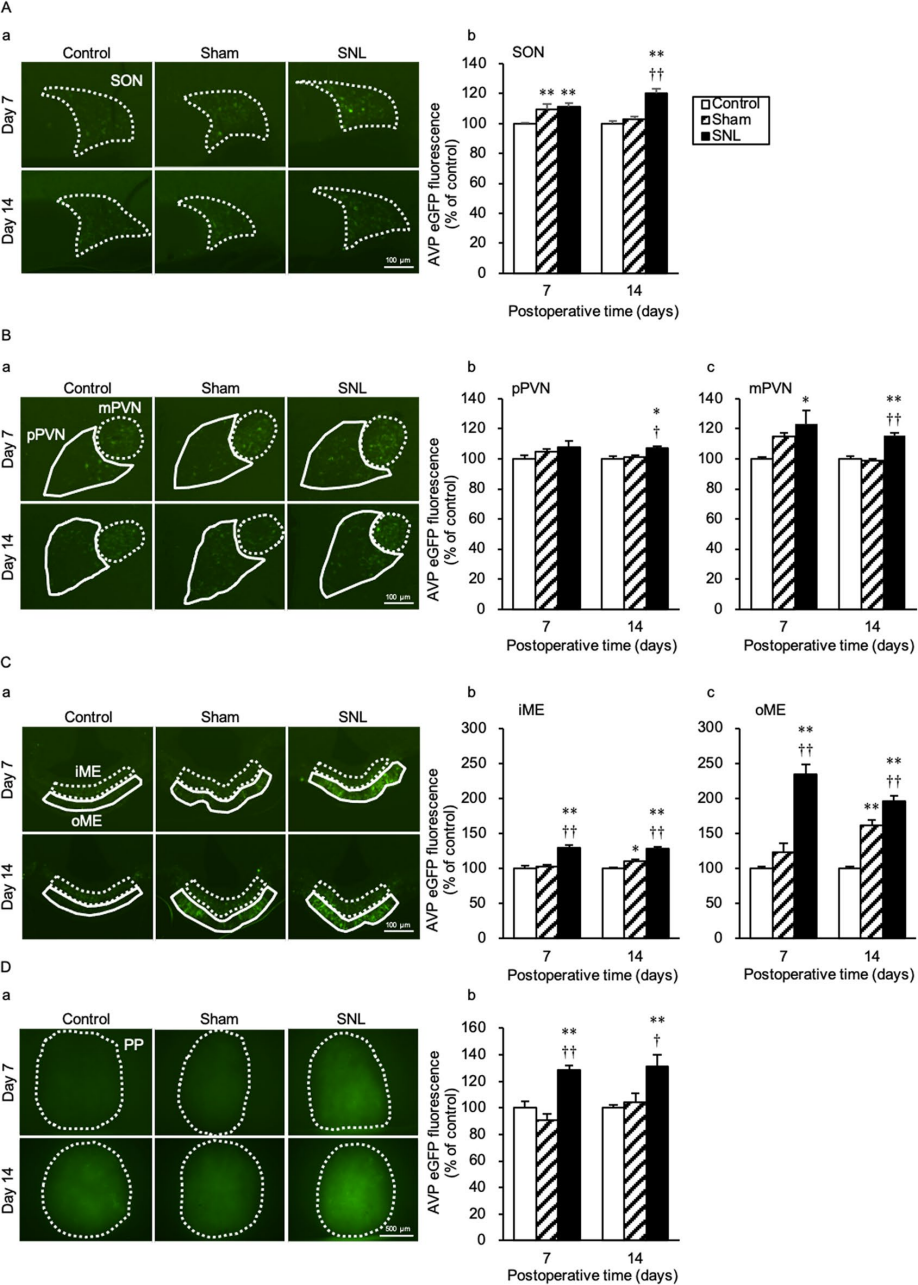
SNL increases intensities of AVP-eGFP fluorescence in SON, PVN, ME, and PP at day 7 and 14 after SNL. (**A**) (**a**) Fluorescence microscopic images of arginine vasopressin (AVP)-enhanced green fluorescent protein (eGFP) in the supraoptic nucleus (SON), where the region surrounded by a white dotted line indicates the SON. (**b**) Average eGFP fluorescence intensities of the SON. (**B**) (**a**) Fluorescence microscopic images of AVP-eGFP in the paraventricular nucleus (PVN), where the regions surrounded by the white dotted line and a white solid line indicate the parvocellular PVN (pPVN) and magnocellular PVN (mPVN), respectively. Average eGFP fluorescence intensities of (**b**) the pPVN and (**c**) mPVN. (**C**) (**a**) Fluorescence microscopic images of AVP-eGFP in the median eminence (ME), where the regions surrounded by a white dotted line and a white solid line indicate the inner ME (iME) and outer ME (oME), respectively; (**b**) and (**c**) present the average eGFP fluorescence intensities of the iME and oME, respectively. (**D**) (**a**) Fluorescence microscopic images of AVP-eGFP in the posterior pituitary (PP), where the region surrounded by a white dotted line indicates the PP. (**b**) Average eGFP fluorescence intensities of the PP. Data are presented as the mean ± SEM (one-way ANOVA). *n* = 6 per group. *p < 0.05 and **p < 0.01 compared with each Control experiment. ^†^*p* < 0.05 and ^††^*p* < 0.01 compared with each Sham experiment.

AVP-eGFP fluorescence in the SON of the SNL and Sham groups at day 7 after the SNL was significantly higher than that of the Control group; however, there was no significant difference between the SNL and Sham groups [F(2,15) = 7.20; SNL vs Control, *p* = 0.009; Sham vs Control, *p* = 0.027; SNL vs Sham, *p* = 1.000]. AVP-eGFP fluorescence in the SON of the SNL group at day 14 after the SNL was significantly higher than that of the Control and Sham groups [F(2,15) = 23.91; SNL vs Control, *p* < 0.001; Sham vs Control, *p* < 0.001] (Fig. 2Aa,b). AVP-eGFP fluorescence in the parvocellular PVN (pPVN) at day 7 after the SNL remained unchanged. However, AVP-eGFP fluorescence in the pPVN of the SNL group at day 14 after the SNL was significantly higher than that of the Control and Sham groups [F(2,15) = 6.70; SNL vs Control, *p* = 0.012; Sham vs Control, *p* = 0.032] (Fig. 2Ba,b). AVP-eGFP fluorescence in the magnocellular PVN (mPVN) of the SNL group at day 7 after the SNL was significantly higher than that of the Control group [F(2,15) = 5.28; SNL vs Control, *p* = 0.017], whereas that of the SNL group at day 14 was significantly higher than that of the Control and Sham groups [F(2,15) = 35.78; SNL vs Control, *p* < 0.001; SNL vs Sham, *p* < 0.001] (Fig. 2Ba,c). AVP-eGFP fluorescence in the median eminence (ME) of the SNL group was significantly higher than that of the Control and Sham groups at day 7 [internal layer of the ME (iME): F(2,15) = 19.66; SNL vs Control, *p* < 0.001; SNL vs Sham, *p* < 0.001, outer layer of the ME (oME): F(2,15) = 44.06; SNL vs Control, *p* < 0.001; SNL vs Sham, *p* < 0.001] and at day 14 [iME: F(2,15) = 29.16; SNL vs Control, *p* < 0.001; SNL vs Sham, *p* < 0.001, oME: F(2,15) = 60.05; SNL vs Control, *p* < 0.001; SNL vs Sham, *p* = 0.004; Sham vs Control, *p* < 0.001] after the SNL (Fig. 2Ca–c). AVP-eGFP fluorescence in the PP of the SNL group was significantly higher than that of the Control and Sham groups at day 7 [F(2,15) = 18.81; SNL vs Control, *p* = 0.001; SNL vs Sham, *p* < 0.001] and at day 14 [F(2,15) = 7.14; SNL vs Control, *p* = 0.008; SNL vs Sham, *p* = 0.038] after the SNL (Fig. 2Da,b).

### SNL increases *AVP* and *proopiomelanocortin* mRNA and decreases *corticotrophin-releasing hormone* mRNA at day 14 after SNL

**Figure 3:**
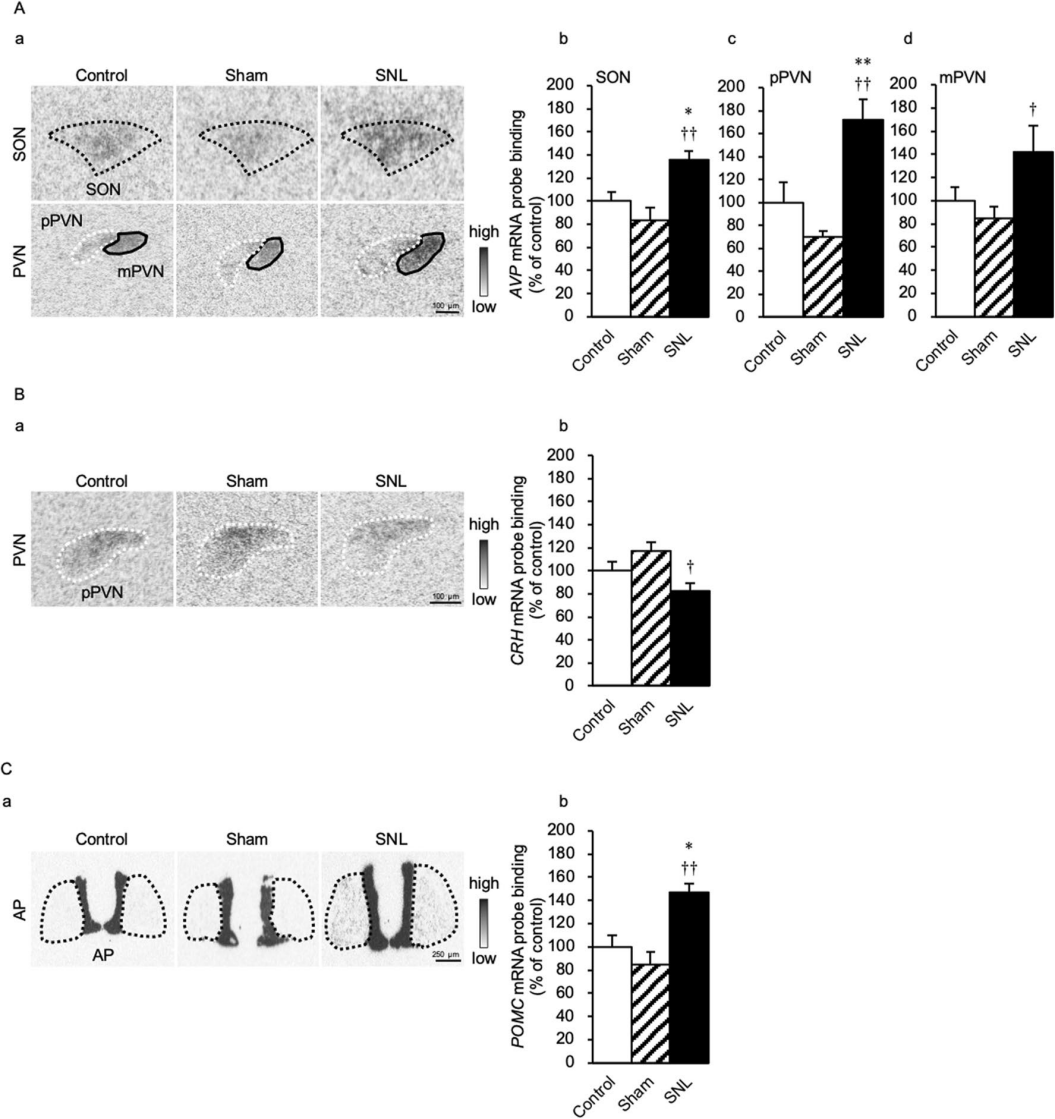
SNL increases the gene expression of *AVP* and *POMC* and decreases *CRH* mRNA levels at day 14 after SNL. (**A**) (**a**) Expression levels of *arginine vasopressin* (*AVP*) mRNA in the supraoptic nucleus (SON) and paraventricular nucleus (PVN). Regions surrounded by the black and white dotted lines and black solid line indicate the SON, the parvocellular PVN (pPVN) and the magnocellular PVN (mPVN), respectively. *AVP* mRNA probe binding (% of control) in (**b**) the SON, (**c**) pPVN, and (**d**) mPVN. (**B**) (**a**) Expression level of *corticotrophin-releasing hormone* (*CRH*) mRNA in the pPVN. Region surrounded by a white dotted line indicates the pPVN. (**b**) *CRH* mRNA probe binding (% of control) in the pPVN. (**C**) (**a**) Expression level of *proopiomelanocortin* (*POMC*) mRNA in the anterior pituitary (AP). Region surrounded by the black dotted line indicates the AP. (**b**) *POMC* mRNA probe binding (% of control) in AP. Data are presented as the mean ± SEM (one-way ANOVA). *n* = 5–7 in each group. *p < 0.05 and **p < 0.01 compared with each Control experiment. ^†^*p* < 0.05 and ^††^*p* < 0.01 compared with each Sham experiment.

The gene expression of *AVP* mRNA in the SNL group were significantly increased in the SON [F(2,14) = 9.17; SNL vs Control, *p* = 0.049; SNL vs Sham, *p* = 0.003], pPVN [F(2,15) = 13.80; SNL vs Control, *p* = 0.009; SNL vs Sham, *p* < 0.001], and mPVN [F(2,15) = 3.85; SNL vs Sham, *p* = 0.046] compared with those in the Control and Sham groups (Fig. 3Aa–d). The gene expression of *corticotrophin-releasing hormone* (*CRH*) mRNA in the SNL group was significantly lower in the pPVN compared with that in the Sham group [F(2,17) = 5.61; SNL vs Sham, *p* = 0.011] (Fig. 3Ba,b). The gene expression of *proopiomelanocortin* (*POMC*) mRNA in the SNL group was significantly increased in the anterior pituitary (AP) compared with that in the Control and Sham groups [F(2,15) = 11.50; SNL vs Control, *p* = 0.010; SNL vs Sham, *p* = 0.001] (Fig. 3Ca,b).

### Mechanical and thermal nociceptive thresholds are elevated at 90 min after CNO administration

**Figure 4:**
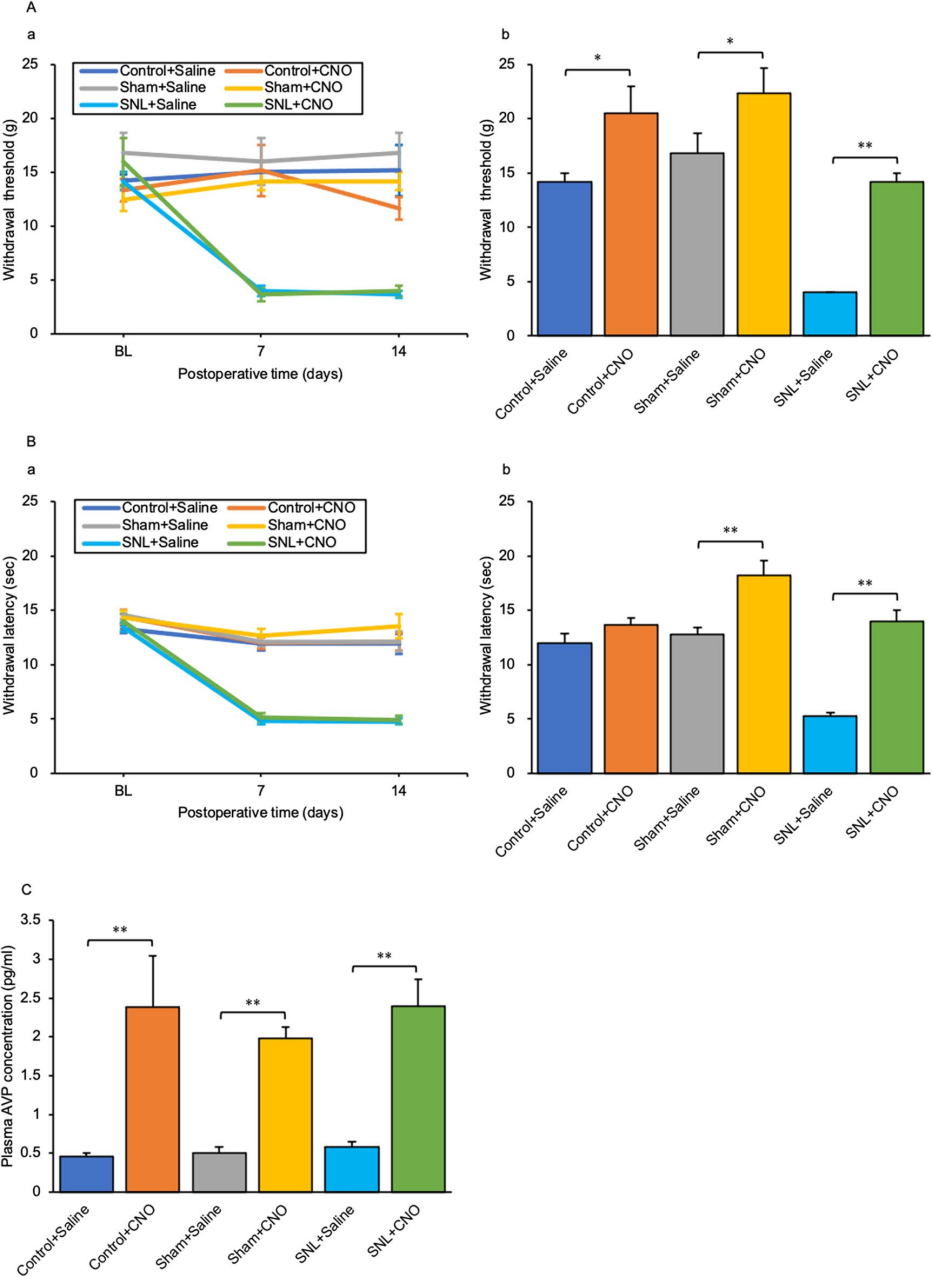
Mechanical and thermal nociceptive thresholds were elevated at 90 min after CNO administration in AVP-hM3Dq-mCherry Tg rats. Changes in (**A**) (**a**) mechanical and (**B**) (**a**) thermal nociceptive thresholds after the SNL procedure. (**A**) (**b**) Mechanical nociceptive threshold 90 min after CNO or saline intraperitoneal (i.p.) injection in each group. (**B**) (**b**) Thermal nociceptive threshold 90 min after CNO or saline i.p. injection in each group. (**C**) Plasma arginine vasopressin (AVP) concentration. Data are presented as the mean ± SEM (Student’s *t*-test). *n* = 6 per group. *p < 0.05 and **p < 0.01 compared with each Saline experiment.

Mechanical and thermal hyperalgesia occurred in all rats in the SNL group on days 7 and 14 after SNL surgery (Fig. 4Aa,Ba). The chemogenetic activation of AVP neurones significantly increased the withdrawal threshold at 90 min after CNO i.p. injection in each CNO i.p. injection group when compared with each Saline i.p. injection group (Control + Saline vs Control + CNO, *p* = 0.018; Sham + Saline vs Sham + CNO, *p* = 0.046; SNL + Saline vs SNL + CNO, *p* < 0.001) (Fig. 4Ab). The activation of AVP neurones after CNO administration significantly prolonged withdrawal latency at 90 min after CNO i.p. injection in each CNO i.p. injection group when compared with each Saline i.p. injection group (Control + Saline vs Control + CNO, *p* = 0.081; Sham + Saline vs Sham + CNO, *p* = 0.003; SNL + Saline vs SNL + CNO, *p* < 0.001) (Fig. 4Bb). The plasma AVP concentration at 90 min after CNO i.p. injection was significantly increased in each CNO group compared with each Saline i.p. injection group (Control + Saline vs Control + CNO, *p* = 0.008; Sham + Saline vs Sham + CNO, *p* < 0.001; SNL + Saline vs SNL + CNO, *p* < 0.001) (Fig. 4C).

### Time course of mechanical nociceptive thresholds after CNO administration and effect of the AVP V1a receptor antagonist

**Figure 5:**
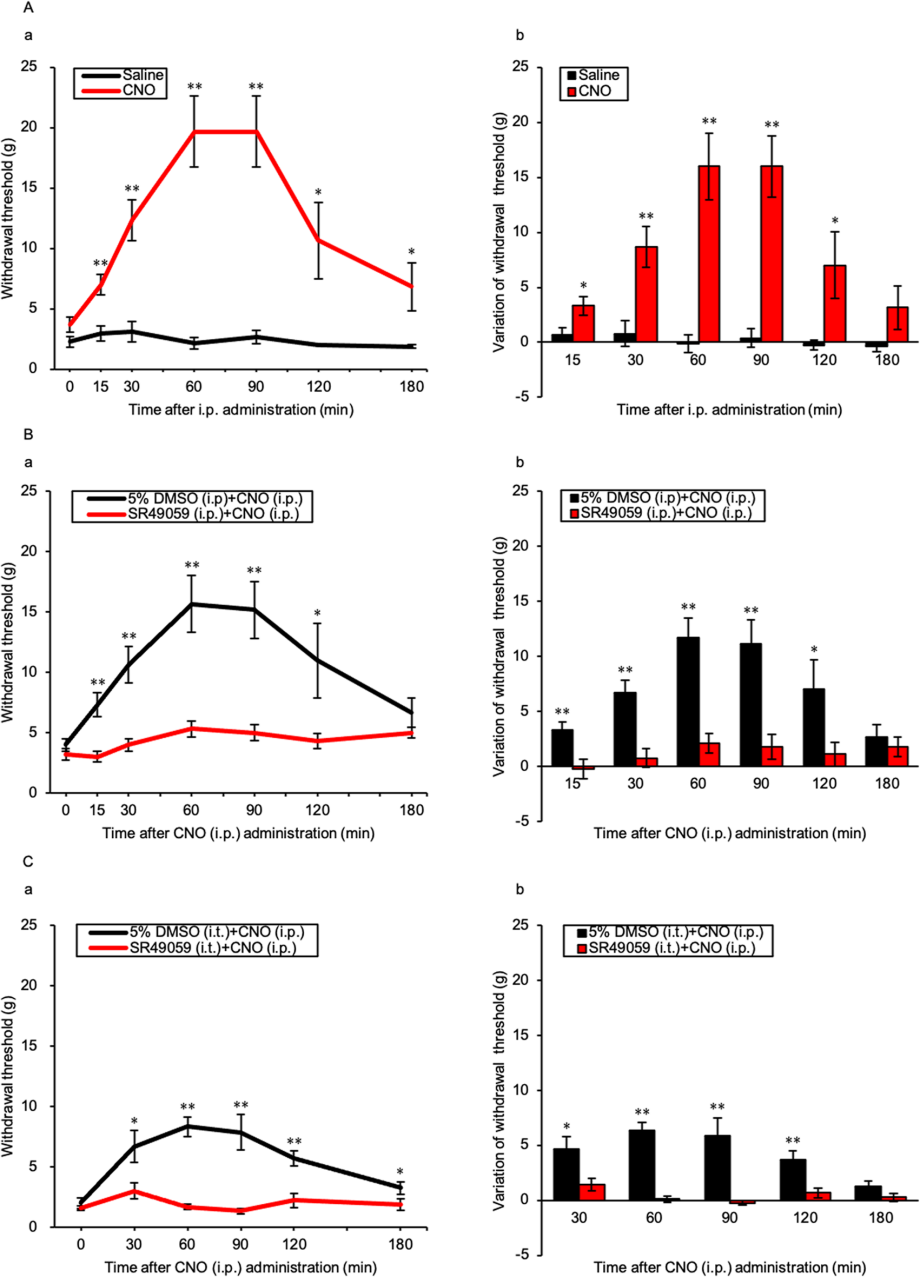
Time course of mechanical nociceptive threshold in AVP-hM3Dq-mCherry Tg rats after CNO administration and effect of the AVP V1a receptor antagonist. (**A**) (**a**) Time course and (**b**) variation of mechanical nociceptive threshold after CNO intraperitoneal (i.p.) injection. Data are presented as the mean ± SEM (Student’s *t*-test). *p < 0.05 and **p < 0.01 compared with each Saline experiment. (**B**) (**a**) Time course and (**b**) variation of mechanical nociceptive threshold after CNO i.p. injection with SR49059 pre-treatment or 5% DMSO i.p. injection. (**C**) (**a**) Time course and (**b**) variation of mechanical nociceptive threshold after CNO i.p. injection with SR49059 pre-treatment or 5% DMSO intrathecal (i.t.) injection. Data are presented as the mean ± SEM (Student’s *t*-test). n = 5–6 in each group. *p < 0.05 and **p < 0.01 compared with each 5% DMSO experiment.

All AVP-hM3Dq-mCherry Tg rats that underwent SNL surgery developed mechanical hyperalgesia. The withdrawal threshold was significantly elevated in the CNO injection group at 15, 30, 60, 90, 120 and 180 min compared with that in the Saline injection group (*p* = 0.003, < 0.001, < 0.001, < 0.001, 0.018, and 0.025, respectively) (Fig. 5Aa). Variations in the withdrawal threshold after administration of CNO were significantly increased at each time point from 15 to 120 min compared with each baseline (*p* = 0.021, 0.004, < 0.001, < 0.001, and 0.030 at 15, 30, 60, 90, and 120 min, respectively) (Fig. 5Ab). Pre-treatment with i.p. injection of SR49059 prevented elevation of the mechanical nociceptive threshold from 15 to 120 min after CNO administration compared with the 5% dimethyl sulfoxide (DMSO) i.p. group (*p* = 0.001, < 0.001, < 0.001, 0.001, and 0.030 at 15, 30, 60, 90, and 120 min, respectively) (Fig. 5Ba). There were statistically significant differences in the variations of withdrawal threshold from 15 to 120 min between the 5% DMSO i.p. and SR49059 i.p. groups (*p* = 0.004, 0.001, < 0.001, 0.002, and 0.036 at 15, 30, 60, 90, and 120 min, respectively) (Fig. 5Bb). Pre-treatment with i.t. injection of SR49059 ablated elevation of the mechanical nociceptive threshold from 30 to 180 min (*p* = 0.017, < 0.001, < 0.001, 0.001, and 0.034 at 30, 60, 90, 120, and 180 min, respectively) (Fig. 5Ca). There were statistically significant differences in the variations of withdrawal threshold between the 5% DMSO i.t. group and the SR49050 i.t. group from the baseline from 30 to 120 min after CNO i.p. injection (*p* = 0.013, < 0.001, 0.002, and 0.005, at 30, 60, 90, and 120 min, respectively) (Fig. 5Cb).

### Time course of thermal nociceptive thresholds after CNO administration and effect of the AVP V1a receptor antagonist

**Figure 6:**
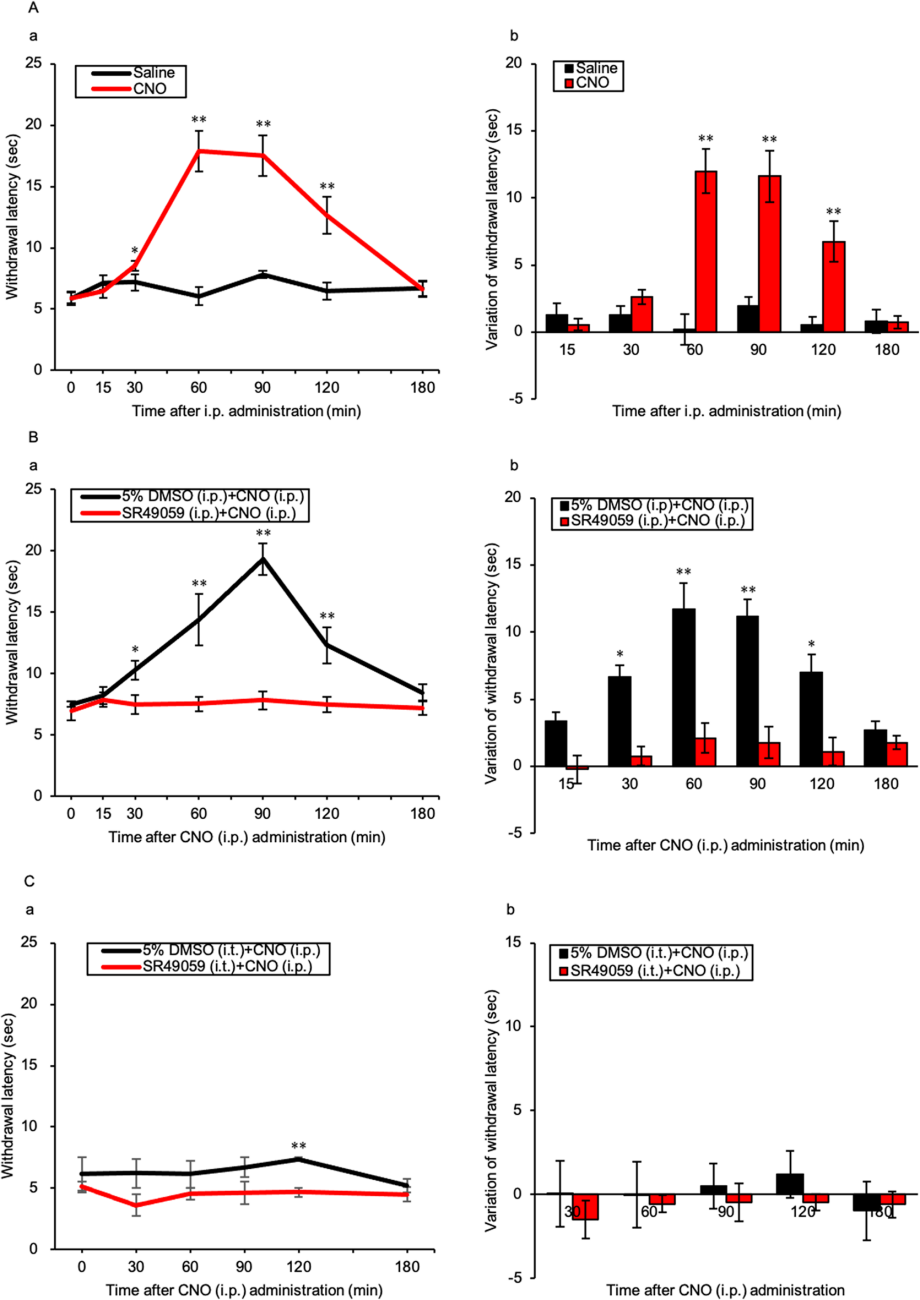
Time course of thermal nociceptive threshold in AVP-hM3Dq-mCherry Tg rats after CNO administration and effect of the AVP V1a receptor antagonist. (**A**) (**a**) Time course and (**b**) variation of thermal nociceptive threshold after CNO or saline intraperitoneal (i.p.) injection. Data are presented as the mean ± SEM (Student’s *t*-test). *p < 0.05 and **p < 0.01 compared with each Saline experiment. (**B**) (**a**) Time course and (**b**) variation of thermal nociceptive threshold after CNO i.p. injection with SR49059 pre-treatment or 5% DMSO i.p. injection. (**C**) (**a**) Time course and (**b**) variation of thermal nociceptive threshold after CNO i.p. injection with SR49059 pre-treatment or 5% DMSO intrathecal (i.t.) injection. Data are presented as the mean ± SEM (Student’s *t*-test). n = 5–6 in each group. *p < 0.05 and **p < 0.01 compared with each 5% DMSO experiment.

All AVP-hM3Dq-mCherry Tg rats that underwent SNL surgery developed thermal hyperalgesia. The withdrawal latency of the CNO injection group was significantly prolonged, from 30 to 120 min, compared with that of the Saline group (*p* = 0.048, < 0.001, < 0.001, and 0.004 at 30, 60, 90, and 120 min, respectively) (Fig. 6Aa). Variations of the withdrawal latency from the baseline were significantly increased from 60 to 120 min after i.p. administration of CNO (*p* = < 0.001, < 0.001, and 0.003 at 60, 90, and 120 min, respectively) (Fig. 6Ab). SR49059 i.p. injection before CNO i.p. injection significantly blocked the analgesic effect of AVP neurone activation from 30 to 120 min after i.p. administration of CNO compared with the 5% DMSO i.p. group (*p* = 0.015, 0.005, < 0.001, and 0.007 at 30, 60, 90, and 120 min, respectively) (Fig. 6Ba). There were statistically significant differences in the variations of withdrawal latency from 30 to 120 min between the 5% DMSO i.p. and SR49059 i.p. groups (*p* = 0.038, 0.001, < 0.001, and 0.015 at 30, 60, 90, and 120 min, respectively) (Fig. 6Bb). There was only a significant difference between the 5% DMSO i.t. and SR49050 i.t. groups at 120 min after i.p. administration of CNO (*p* = < 0.001) (Fig. 6Ca). There was no significant difference in the variation of withdrawal latency between the 5% DMSO i.t. group and the SR49050 i.t. group from 30 to 180 min after CNO i.p. injection (Fig. 6Cb).

### AVP neurones activate serotonergic neurones in the DR, noradrenergic neurones in the LC and paired box gene-2 positive inhibitory interneurones in the spinal dorsal horn

**Figure 7:**
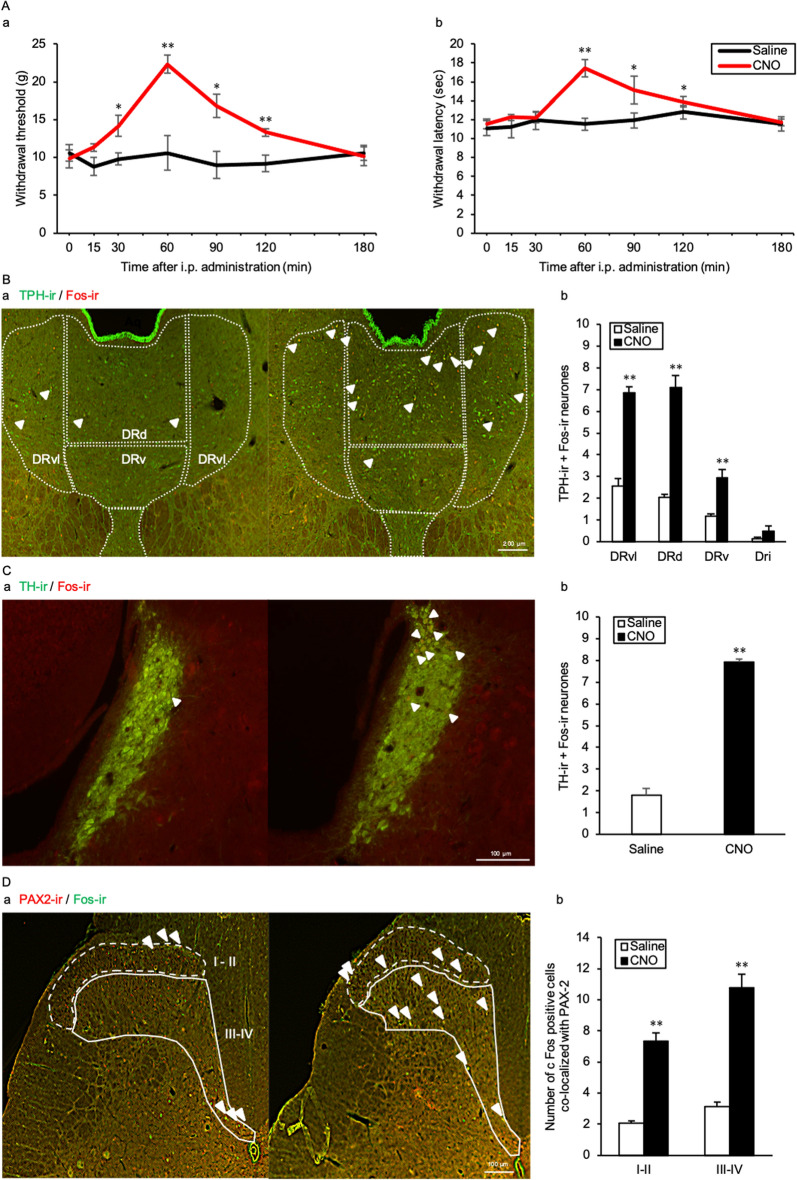
AVP neurones activate serotonergic neurones in the DR, noradrenergic neurones in the LC and PAX-2 positive interneurones of the spinal dorsal horn. (**A**) Time course of (**a**) mechanical and (**b**) thermal nociceptive threshold after CNO or saline intraperitoneal (i.p.) injection. Data are presented as the mean ± SEM (Student’s *t*-test). *p < 0.05 and **p < 0.01 compared with each Saline experiment. (**B**) (**a**) Fluorescence microscopic images of tryptophan hydroxylase (TPH)-ir (green) and Fos-ir (red) positive neurones in the dorsal raphe (DR). (**b**) The number of TPH-ir and Fos-ir positive neurones in the DR. Data are presented as the mean ± SEM (Student’s *t*-test). *p < 0.05 and **p < 0.01 compared with each Saline. (**C**) (**a**) Fluorescence microscopic images of tyrosine hydroxylase (TH)-ir (green) and Fos-ir (red) positive neurones in the locus coeruleus (LC). The number of TH-ir and Fos-ir positive neurones in the LC. Data are presented as the mean ± SEM (Student’s *t*-test). **p < 0.01 compared with Saline. The fluorescence microscopic images of paired box gene-2 (PAX-2)-ir (red) and Fos-ir (green) positive cells in the lumber segment 5 (L5) ipsilateral dorsal horn. (**b**) The number of PAX-2-ir and Fos-ir positive cells in the L5 spinal dorsal horn. Data are presented as the mean ± SEM (Student’s *t*-test). n = 6 in each group. **p < 0.01 compared with Saline.

The withdrawal threshold of the CNO group was significantly elevated, from 30 to 120 min, compared with that of the Saline group in untreated AVP-hM3Dq-mCherry Tg rats (*p* = 0.009, 0.001, 0.006, and 0.005 at 30, 60, 90, and 120 min, respectively) (Fig. 7Aa). The withdrawal latency of the CNO injection group was significantly prolonged from 60 to 120 min, compared with that of the Saline group (*p* = < 0.001, 0.048, and 0.001 at 60, 90, and 120 min, respectively) (Fig. 7Ab). The number of tryptophan hydroxylase (TPH)- and Fos-ir double positive neurones were significantly increased in the CNO group in the ventrolateral part of DR (DRvl), the dorsal part of DR (DRd), ventral part of DR (DRv), compared with the Saline group (DRvl, *p* < 0.001; DRd, *p* < 0.001; DRv, *p* < 0.001) (Fig. 7Ba,b). The tyrosine hydroxylase (TH)- and Fos-ir double positive neurones were significantly increased in the CNO group in the LC, compared with the Saline group (*p* < 0.001) (Fig. 7Ca,b). The number of paired box gene-2 (PAX-2) and Fos-ir colocalized neurones were significantly increased in the CNO group in the laminae I–II and III–IV of L5 ipsilateral dorsal horn, compared with the Saline group (laminae I–II, *p* < 0.001; laminae III–IV, *p* < 0.001) (Fig. 7Da,b).

## Discussion

The results of this study are summarised as follows: (1) the fluorescence intensity of eGFP in the SNL group was significantly increased in the SON, PVN, ME, and PP; (2) the expression level of *AVP* mRNA in the SON and PVN and *POMC* mRNA in the AP was significantly increased, whereas that of *CRH* mRNA in the pPVN was significantly decreased; (3) the activation of endogenous AVP neurones by CNO administration led to an analgesic effect; (4) the analgesic effect of endogenous AVP was significantly suppressed by pre-treatment with a V1a receptor antagonist; (5) serotonergic neurones in the DR, noradrenergic neurones in the LC and PAX-2 positive interneurones in the spinal dorsal horn were found to be activated after stimulation of endogenous AVP neurones.

AVP produced in the mPVN and SON is transported to the PP via the inner layer of the median ridge and secreted into the systemic circulation. Despite increased AVP-eGFP and *AVP* mRNA expression in the magnocellular region, the plasma AVP concentration was not significantly increased (see Supplementary Fig. S1 online), and mechanical and thermal hyperalgesia were still observed in the SNL. Although the expression of AVP-eGFP, *AVP* mRNA, and plasma AVP concentration was significantly increased, hyperalgesia persisted in our previous AA model and knee OA model^20,22^. We previously reported that AVP and Oxytocin (OXT) exhibited similar kinetics in various pain models^20,21,25^. OXT-mRFP1 fluorescence intensity in the SON, mPVN and PP was significantly increased in the NP model rats using OXT-mRFP1 Tg rats in which OXT was labelled with red fluorescent protein, but plasma OXT concentration remained unchanged^26^. The half-life of blood AVP and OXT is very short and their plasma concentrations may not correlate with their activity in the brain^27^. Therefore, it seems possible that NP activates the synthesis of AVP without stimulating its secretion. The SNL group exhibited a significant increase in the fluorescence intensity of AVP-eGFP and *AVP* mRNA expression in the pPVN, and *CRH* mRNA levels in the pPVN were significantly decreased at day 14 after SNL. AVP and CRH secreted from the parvocellular region of the PVN are secreted from axon terminals in the outer layer of the median ridge to the pituitary portal vein. Secretion of adrenocorticotropic hormone (ACTH) from the AP activates the hypothalamo-pituitary-adrenal (HPA) axis^28^. CRH and AVP have a secretagogue effect on the ACTH and work synergistically in the HPA axis^29^. In a previous study, chronic pain induced by AA displayed dysfunction of the HPA axis^30,31^. Indeed, AVP, but not CRH, plays a key role in promoting ACTH secretion under chronic stress conditions^30^. In our previous study, *AVP* mRNA levels in the pPVN were significantly increased, and *CRH* mRNA was significantly decreased in a chronic pain model of AA^20^. In the present study, we showed that AVP might be a key modulator in the HPA axis of the NP model.

SR49059 is a V1a receptor antagonist, but may also act on V1b and OXT receptors. It was reported that SR49059 had an affinity more than two orders of magnitude lower for V1b and OXT receptors and did not exhibit intrinsic agonist activity^32^. On the other hand, it has been reported that 3–5% of neurones in the magnocellular of the hypothalamus co-express AVP and OXT^33^. Our previous report also showed OXT immunostaining positivity in SON (3.57%) and PVN (2.17%) mCherry neurones in AVP-hM3Dq-mCherry Tg rats^24^. Hence, i.p. CNO administration may activate some OXT neurones in the hypothalamus, which may be involved in pain modulation. However, although the plasma OXT concentration was not measured in the present study, the proportion of neurones co-expressing OXT and AVP was only a few percent, which suggests that the effect may be small. Accordingly, our data suggest that endogenous AVP is involved in pain modulation by central and peripheral V1a receptors. In the central nervous system, microinjection of AVP into the amygdala, caudate nucleus, and nucleus accumbens increases pain thresholds^34–36^. It has also been suggested that the analgesic mechanism of AVP in the central nervous system may be involved in endogenous opioid and serotonin systems^37^. In addition, a projection from the hypothalamus to the spinal dorsal horn involved in analgesia via V1a receptors is thought to exist^38^. In the peripheral nervous system, a previous study showed that AVP upregulates the function of GABA_A_ in dorsal root ganglion neurones^39^. Furthermore, DNA microarray analysis revealed that the V1a receptor was upregulated in a mouse model of NP^40^. Subcutaneous plantar injection of AVP was shown to attenuate formalin-induced nociceptive pain via peripheral V1a receptors^6^.

Furthermore, the activation of endogenous AVP neurones increased the neuronal activity of the serotonergic DR neurones, noradrenergic LC neurones and inhibitory interneurones of the spinal dorsal horn involved in the descending pain inhibitory system. The DR and LC are important neuronal nuclei involved in the DPMS and these nuclei project to the spinal dorsal horn^15,41–44^. The activation of the DR and LC releases serotonin and noradrenaline, respectively^41^. AVP neurones project to the DR and activate serotonergic neurones via V1a receptors^45,46^. Moreover, AVP neurones also project to the LC, and V1a and V1b receptors are located on noradrenergic neurones, resulting in noradrenergic neuronal activation^14,45,47^. Thus, chemogenetic activation of AVP neurones in AVP-hM3Dq-mCherry-Tg rats may activate serotonergic and noradrenergic neurones, which in turn activate PAX-2 positive inhibitory interneurones in the spinal dorsal horn.

This study has several limitations. AVP neurones have been located in the central nervous system in the piriform cortex, olfactory bulb, retina, and LC using AVP-eGFP Tg rats^48^. Administration of CNO could also activate AVP neurones in these regions, but we have not confirmed in the present study whether hM3Dq is expressed in regions other than the hypothalamus. However, the main source of AVP production is likely to be the hypothalamic SON, PVN, and suprachiasmatic nucleus (SCN)^48^. AVP production by non-hypothalamic AVP neurones is considered to be small. Also, we did not perform CNO administration experiments using Wistar rats that do not express hM3Dq. Clozapine is the parent compound of CNO, which has been demonstrated to be converted to clozapine^49^. I.p. administration of clozapine (30 mg/kg) was reported to activate hypothalamic vasopressin neurones^50^. In our previous studies, it was confirmed that i.p. administration of CNO (1 mg/kg) to Wistar rats did not significantly increase the number of c-Fos positive neurones in the SON and PVN, nor plasma AVP concentrations^24^. Therefore, the pharmacological action of CNO itself on the AVP system is considered to be poor in rats that do not express hM3Dq. The c-Fos positive neurones in the DR and LC may contain fibres of the ascending pain modulation system; however, it is difficult to distinguish between neurones of the ascending pain modulation system and those of the DPMS. Nevertheless, our study showed that the number of inhibitory interneurone-specific PAX-2 positive neurones in the spinal dorsal horn, which is the end point of the descending pathway, was increased, which may indicate that chemogenetic activation of endogenous AVP neurones stimulated the DPMS. Besides, PAX-2-positive neurones may be activated by pathways other than the descending pain-inhibitory system, such as the dorsal root ganglia. Further studies using AVP-hM3Dq-mCherry Tg rats are required.

In conclusion, the results of this study suggest that the hypothalamo-neurohypophysial system of AVP is upregulated in a rat model of NP and that activated endogenous AVP exerts an analgesic effect via the V1a receptor and may activate the DPMS.

## Methods

### Animals

AVP-eGFP Tg rats (7 weeks old, weighing 238–305 g) and AVP-hM3Dq-mCherry Tg rats (7 weeks old, weighing 253–350 g) were bred and maintained as described previously^22,24,51^. All procedures were performed according to the guidelines on the use and care of laboratory animals established by the Physiological Society of Japan. All experiments performed in this study were approved by the Ethics Committee on Animal Care and Experimentation (permission number: AE10-012) of the University of Occupational and Environmental Health, Japan. This research was conducted in accordance with ARRIVE guidelines.

### Test substances

CNO (Sigma-Aldrich Japan Co. LLC., Tokyo, Japan) was dissolved in saline (Otsuka Pharmaceutical Co. Ltd., Tokyo, Japan) at a previously determined dosage (1 mg/kg)^24^. The solubility of SR49059 (Cayman Chemical, Ann Arbor, MI, USA) was 5 mg/1 mL. Ten milligrams of SR49059 was dissolved in 2 mL 100% DMSO and diluted with saline to make SR49059 in 5% DMSO solution. The i.p. (1 mg/kg) and i.t. dosage of SR49059 (12 nmol/30 μL) were determined in previous studies^52,53^.

### Spinal nerve ligation procedure

AVP-eGFP Tg and AVP-hM3Dq-mCherry Tg rats were randomly divided into three groups: Control, Sham and SNL. Rats in the Control group were untreated, whereas rats in the sham surgery (Sham) group were anaesthetised through subcutaneous injection with a combination of anaesthetics (0.3 mg/kg of medetomidine, 4.0 mg/kg of midazolam, and 5.0 mg/kg of butorphanol), and the left lumbar 5 (L5) spinal nerve was disclosed without ligation. Rats in the SNL model group were anaesthetised, and the left L5 spinal nerve was ligated using 6-0 silk sutures^54^.

### Measurement of mechanical and thermal sensitivities

The mechanical and thermal nociceptive thresholds were evaluated at the baseline (BL, before the SNL procedure) and at postoperative days 7 and 14 in AVP-eGFP Tg rats. Before evaluating the mechanical and thermal nociceptive thresholds, rats were acclimatised to the experimental environment for at least 30 min. Thereafter, we performed the manual von Frey test on the left hind paw using calibrated von Frey filaments (North Coast Medical, Gilroy, CA, USA) and a hot plate test (52.5 °C) as described previously^22^. The mechanical and thermal nociceptive thresholds were determined according to previously described methods^21,22,26^.

### Tissue preparation

At postoperative days 7 and 14 after the SNL or sham surgery, AVP-eGFP Tg rats were anaesthetised through subcutaneous injection with a combination of anaesthetics, described in “Spinal nerve ligation procedure”. Perfusion using 0.1 M phosphate buffer (PB; pH 7.4) with heparin (1000 U/L) and 4% paraformaldehyde in 0.1 M PB was performed transcardially. The brain, pituitary, and spinal cords were gently removed and post-fixed with 4% paraformaldehyde in 0.1 M PB for 48 h at 4 °C, then cryoprotected in 20% sucrose in 0.1 M PB for 48 h at 4 °C^21,22,26,51^. The fixed brain and spinal cord were cut into a thickness of 30 μm using a microtome (Yamato Kohki Industrial Co. Ltd, Saitama, Japan). Each section of the SON, PVN, ME, PP, DR, LC, and L5 spinal dorsal horn was selected according to “The Rat Brain in Stereotaxic Coordinates”^55^.

### Evaluation of glial cells in the spinal dorsal horn using immunohistochemistry

Three sections of the spinal cord were rinsed twice with 0.1 M phosphate-buffered saline (PBS) and washed in 0.1 M Tris buffer (pH 7.6) containing 0.3% Triton X-100^45^. For fluorescence immunohistochemistry of ionised calcium-binding adapter molecule-1 (Iba-1, marker of microglia) and the glial fibrillary acidic protein (GFAP, marker of astrocyte), sections of the L5 of the spinal cord were incubated for 3 days at 4 °C in a rabbit primary anti-Iba1 antibody solution (1:2000; Wako Pure Chemical Industries, Ltd, Osaka, Japan) or a rabbit primary anti-GFAP antibody solution (1:1000; Sigma-Aldrich Japan Co. LLC). After a double wash treatment in 0.3% Triton X-100 in PBS, samples were subsequently incubated with a secondary antibody solution (Alexa Fluor 546-conjugated anti-rabbit IgG antibody raised in goat; Molecular Probes, Eugene, OR, USA; 1:1000; dissolved in 0.1 M PBST) for 2 h at room temperature. Sections were washed thrice in 0.1 M PBS for a total of 30 min and mounted on glass slides for observation using a fluorescence microscope with an RFP filter (Nikon Corporation, Tokyo, Japan). Digital images were captured using a digital camera (DS-Qi1Mc; Nikon Corporation). The ipsilateral L5 laminae I and II regions of interest were manually surrounded, and the averaged values of the Iba-1 and GFAP fluorescence intensities were analysed using NIS Elements microscope imaging software (Nikon Corporation).

### Expression level of AVP-eGFP in the SON, PVN, ME, and PP

Digital images of the SON, pPVN, mPVN, ME, PP, and spinal cord were captured using fluorescence microscopy with a GFP filter (ECLIPS E 600; Nikon Corporation) to measure the fluorescence intensities of AVP-eGFP^22^. Two sections, each including SON, PVN, and ME, were selected for image analysis. We calculated the mean fluorescence intensity value for the eGFP per unit area in the SON, pPVN, mPVN, iME, oME, and PP for each animal group on postoperative days 7 and 14 using NIS Elements software (Nikon Corporation).

### In situ hybridisation histochemistry for *AVP*, *CRH*, and *POMC* mRNA

AVP-eGFP Tg rats (*n* = 7 in each group) were decapitated at postoperative day 14 for in situ hybridisation histochemistry. The brains obtained after decapitation were placed in a deep freezer at − 80 °C. Coronal sections of the brains were cut into a thickness of 12 μm using a cryostat (OTF5000, Bright Instrument Co, Ltd., England) at − 20 °C and mounted onto glass slides coated with gelatin/chrome alum. Two sections of the SON and PVN, and six sections of the AP were selected to measure the density of the autoradiograph. Subsequently, we used a ^35^S-labelled oligodeoxynucleotide probe for *AVP*, *CRH*, and *POMC* mRNA analysis^56^. The autoradiographic images for the two selected sections of *AVP* mRNA in the SON and PVN, *CRH* mRNA in the PVN, and those for the four selected sections of *POMC* mRNA in the AP were captured using a charge-coupled device camera (DAGE-MTI, Michigan City, IN, USA). These images were measured to obtain the mean *AVP* mRNA levels per unit area in the SON, mPVN, and pPVN, the mean *CRH* mRNA levels in the pPVN, and the mean *POMC* mRNA levels in the AP for each animal group at day 14 after SNL surgery using ImageJ software (National Institutes of Health, Bethesda, MD, USA).

### Analgesic effects of endogenous AVP on mechanical and thermal nociceptive thresholds

AVP-hM3Dq-mCherry Tg rats were randomly assigned to Control, Sham, and SNL groups. Mechanical and thermal nociceptive thresholds were evaluated at BL, days 7, 14, and 15 after the SNL surgery. At day 15 after the SNL surgery, we evaluated the nociceptive thresholds 90 min after the i.p. injection of CNO (1 mg/kg) or saline.

### Effects of V1a antagonist on mechanical and thermal nociceptive thresholds

All AVP-hM3Dq-mCherry Tg rats underwent SNL surgery and developed NP. Thereafter, rats were randomly divided into the following groups: CNO i.p., 5% DMSO i.p., SR49059 i.p. + CNO i.p., 5% DMSO i.p. + CNO i.p., SR49059 i.t. + CNO i.p., and 5% DMSO i.t. + CNO i.p. At postoperative day 15, we evaluated the mechanical/thermal nociceptive threshold from the baseline (0 min) to 180 min after i.p. administration of CNO. CNO i.p. injection was performed 5 min after SR49059 or 5% DMSO administration. Variations in the mechanical/thermal threshold from the baseline (0 min) were calculated at each time point.

### Plasma AVP concentrations

We collected the trunk blood of AVP-hM3Dq-mCherry Tg rats decapitated after the behavioural experiment at postoperative day 15 after SNL surgery. Plasma samples were obtained by centrifugation for 10 min at 4 °C for 1000*g* and measured in duplicate. Plasma AVP concentrations were determined by radioimmunoassay with specific anti-AVP antibodies^57^. The results were averaged for each group.

### Evaluation of the activity of serotonergic DR neurones and noradrenergic LC neurones and inhibitory interneurones of the spinal dorsal horn

Untreated AVP-hM3Dq-mCherry Tg rats were divided into the CNO injection group and the Saline injection group. Mechanical and thermal nociceptive thresholds were measured from baseline to 180 min after CNO or saline i.p. administration. Two weeks after evaluation of pain threshold, perfusion fixation was performed at 90 min after CNO or saline injection, and the brain and spinal cord were removed; three sections of the DR and LC and L5 spinal dorsal horn were prepared as described in “Tissue preparation”. For double immunohistochemistry, each three brain sections were incubated for 72 h at 4 °C with a goat primary c-Fos antibody (sc-52G; Santa Cruz Biotechnology, Dallas, TX, USA; 1:250) and a rabbit primary TPH antibody (Bioss Antibodies, Woburn, MA, USA; 1:100) or a rabbit primary TH antibody (GeneTex, Irvine, CA, USA; 1:200) in PBST with 5% normal donkey serum. Three spinal cord slices were incubated with a rabbit primary c-Fos antibody (Santa Cruz Biotechnology, Dallas, TX, USA; 1:1000) and a mouse primary PAX-2 antibody (Abnova, Taipei, Taiwan; 1:500) in PBST with 5% normal donkey serum. After three washes with PBST for a total of 30 min, the slices were incubated for 24 h at 4 °C with a secondary antibody solution. Sections were washed three times in 0.1 M PBS for a total of 30 min. The images of DR and L5 spinal dorsal horn were observed using a BZ-X800 All-in-One fluorescence microscope (Keyence, Osaka, Japan). The sections of LC were observed using Nikon fluorescence microscope. The number of co-expressing neurones in each of the three sections was counted and averaged.

### Statistics and reproducibility

The data are shown as the mean ± standard error of the mean (SEM), and the mean deviation from the control rats (%) ± SEM were calculated from the results. All data were analysed using one-way analysis of variance with Bonferroni’s post hoc test for multiple comparisons. The unpaired Student’s *t*-test was used based on the results of the Shapiro–Wilk test for comparison between the two groups (Stata/IC 16; StataCorp, College Station, TX, USA). Statistical significance was set at *p* < 0.05. Each experiment was carried out independently at least twice, with similar results. The sample size for this experiment was determined on the basis of previous reports which showed significant effects.

## Supplementary Information

Supplementary Figure S1.

## Data Availability

All data generated or analysed during this study are included in this published article (and its Supplementary Information files).
