# Supplementary figures and images for: Upregulation of the hypothalamo-neurohypophysial system and activation of vasopressin neurones attenuates hyperalgesia in a neuropathic pain model rat
Source: Sci Rep. 2022 Jul 29;12:13046. doi: 10.1038/s41598-022-17477-5 (PMC9338054; doi:10.1038/s41598-022-17477-5)

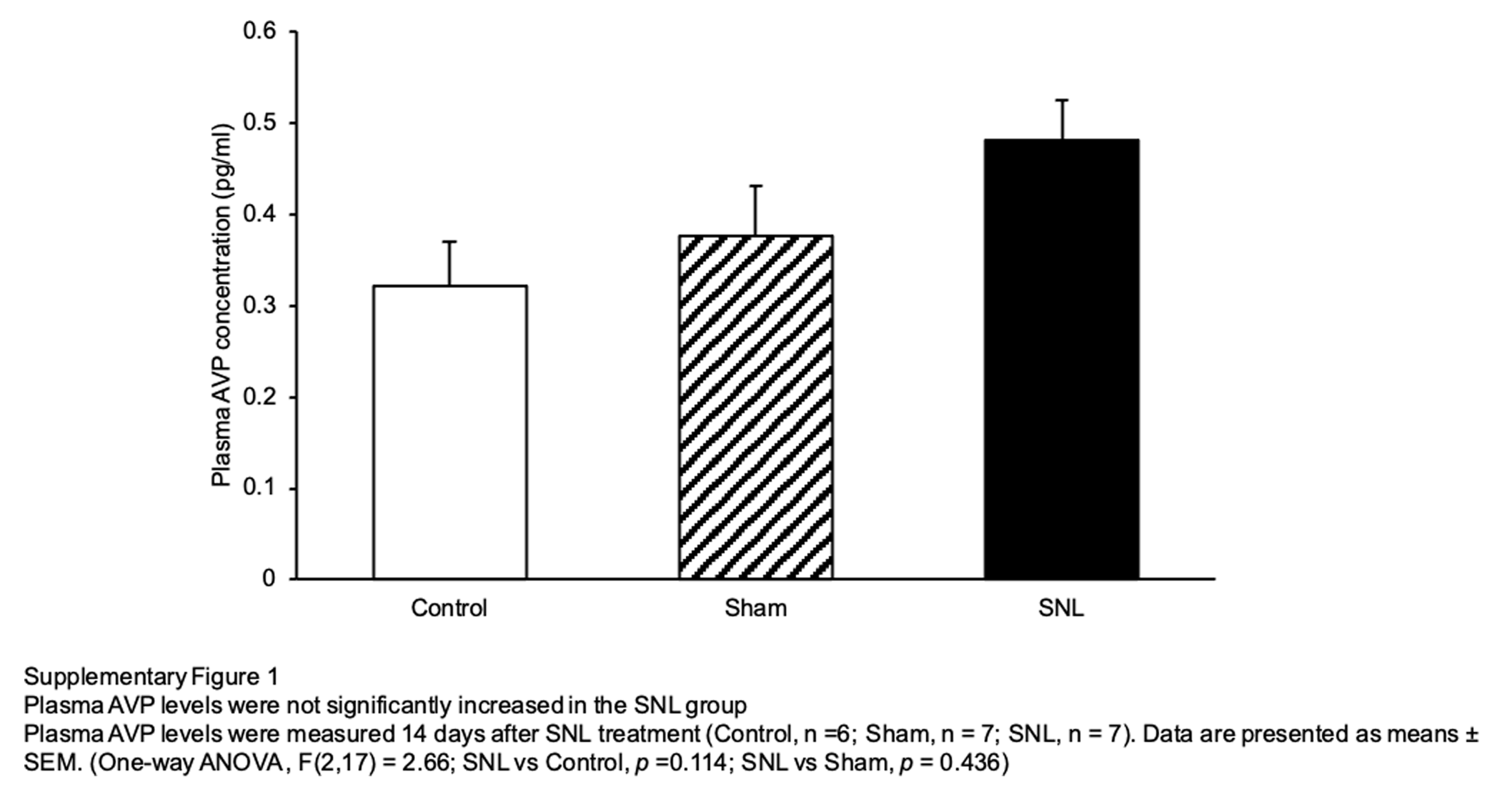

Supplement: Supplementary file 1 — Supplementary Figure S1. [file 41598_2022_17477_MOESM1_ESM.tif]
